# Unilateral sternal head resection in an adult case of congenital torticollis: A rare anatomical variation

**DOI:** 10.1016/j.ijscr.2025.111499

**Published:** 2025-06-10

**Authors:** Seya Arafeh, Roba Alzuhoor, Hind Amleh, Yasmin Bozia, Munther Shawar

**Affiliations:** a Faculty of Medicine, Hebron University, Hebron 150, Palestine; b Al-Hilal specialized Red Crescent hospital, Hebron, Palestine

**Keywords:** Case report, Congenital muscular torticollis, Facial asymmetry, Cheng and Tang scoring system

## Abstract

**Introduction and importance:**

This case report describes the rare diagnosis of congenital muscular torticollis (CMT) in adulthood. While of congenital muscular torticollis (CMT) is commonly identified in infancy, delayed presentation can lead to persistent functional and cosmetic impairments. This case highlights the challenges of late diagnosis and the role of surgical intervention in achieving favorable outcomes.

**Presentation of case:**

A 20-year-old woman presented with restricted neck mobility and facial asymmetry. Clinical examination and imaging confirmed unilateral sternocleidomastoid muscle (SCM) contracture with an absent clavicular head. The patient underwent unilateral sternocleidomastoid muscle (SCM) resection. Postoperative rehabilitation significantly improved mobility and aesthetic concerns.

**Clinical discussion:**

Late-presenting congenital muscular torticollis (CMT) is uncommon and requires individualized management. This case demonstrates how unilateral sternocleidomastoid muscle (SCM) resection can be an effective alternative to traditional bipolar release. Postoperative rehabilitation played a crucial role in restoring function.

**Conclusion:**

This report underscores the potential for successful intervention in neglected congenital muscular torticollis (CMT) cases. Key takeaways include:

Late-presenting congenital muscular torticollis (CMT) can still be effectively treated with surgery and rehabilitation.

Anatomical variations must be considered for optimal surgical planning.

A tailored surgical approach, including unilateral sternocleidomastoid muscle (SCM) resection, can yield significant functional and cosmetic improvements.

## Highlights

• CMT can present in adults, causing cosmetic deformities and restricted mobility.
• Surgery in a 20-year-old revealed a rare unilateral absence of the clavicular head.
• Post-op results showed improved mobility and symmetry, proving surgery's efficacy.
• Rehab after surgery led to excellent functional and cosmetic outcomes.
• This case stresses tailored treatment and documenting rare CMT variations.

## 1 Introduction

Congenital muscular torticollis (CMT) is a condition of shortening and fibrous replacement of the sternocleidomastoid (SCM) muscle that develops successively either at birth or shortly after [1]. The reported incidence ranges from 0.3 % to 2.0 % [2]. The exact cause of Congenital muscular torticollis (CMT) remains unknown, although several theories have been proposed, including ischemia, trauma during delivery, and abnormal positioning in utero [3]. Children with Congenital muscular torticollis (CMT) commonly present with swelling and deformity of the neck in the first week of life [4]. If left untreated, CMT can lead to complications such as pain, spinal deformities, and craniofacial irregularities [5,6].

The objective of this study is to assess the effectiveness of surgical intervention, rehabilitation, and post-operative outcomes in restoring neck mobility and improving cosmetic appearance. It also highlights the role of early diagnosis and intervention. This case report has been reported in line with the SCARE 2023 criteria [7].

## 2 Case presentation

A 20-year-old woman presented with concerns about cosmetic appearance and limited neck motion. At age 17, strangers noticed head deviation and uneven shoulder levels. Despite physiotherapy and dry needling, there was no improvement. At 18, she was diagnosed with congenital torticollis and advised to undergo surgery, though she hesitated due to concerns about incomplete resolution.

The patient experienced neck pain, blurred vision, and mild hearing impairment after attempting corrective exercises. Over time, she developed increased stiffness in the right side of her neck, prominent clavicular bones, and facial asymmetry, especially after sleeping on her right side.

**Fig. 1:**
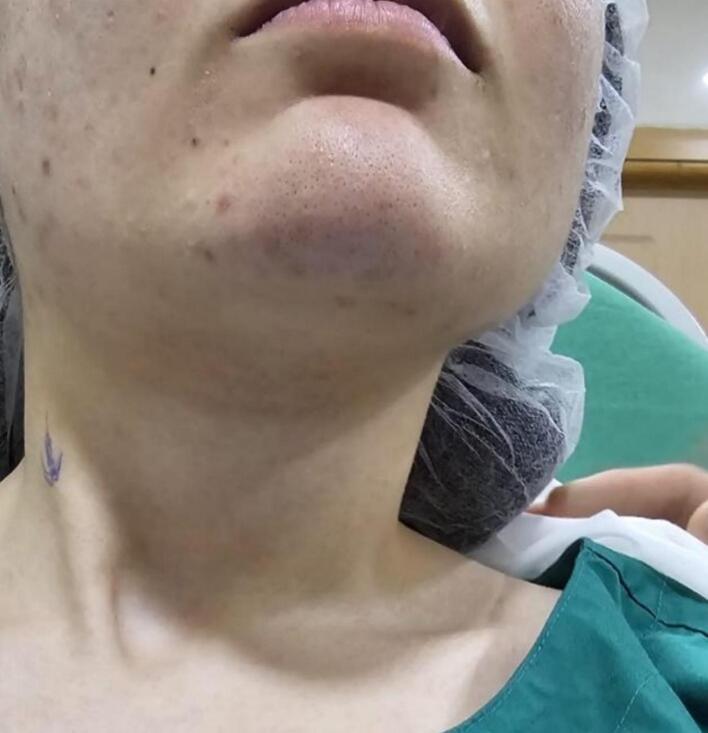
Preoperatively shows facial asymmetry and the cord like structure on the right.

**Fig. 2:**
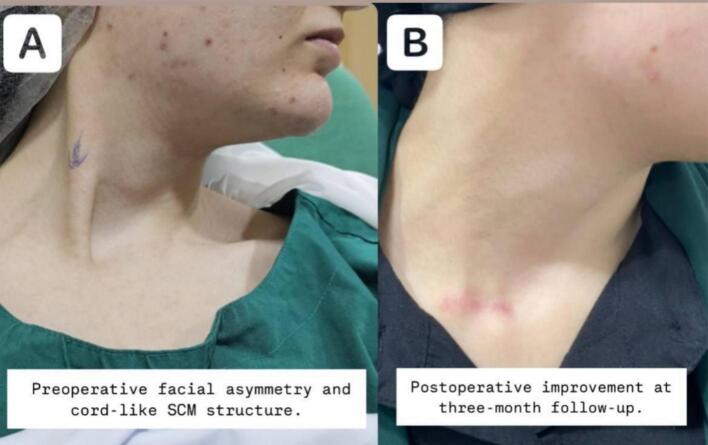
Comparison between the SCM preoperative and postoperative.

**Fig. 3:**
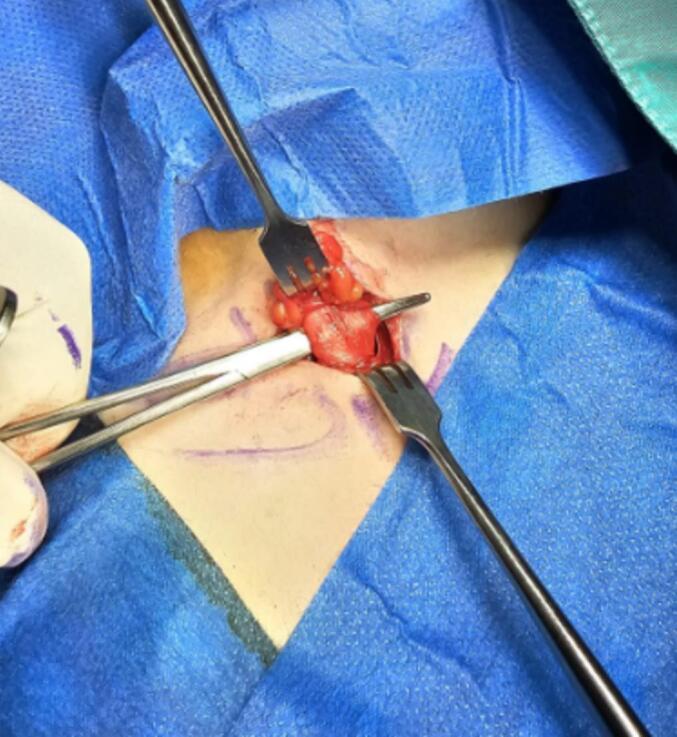
Intraoperative SCM tenotomy, unipolar single approach.

**Fig. 4:**
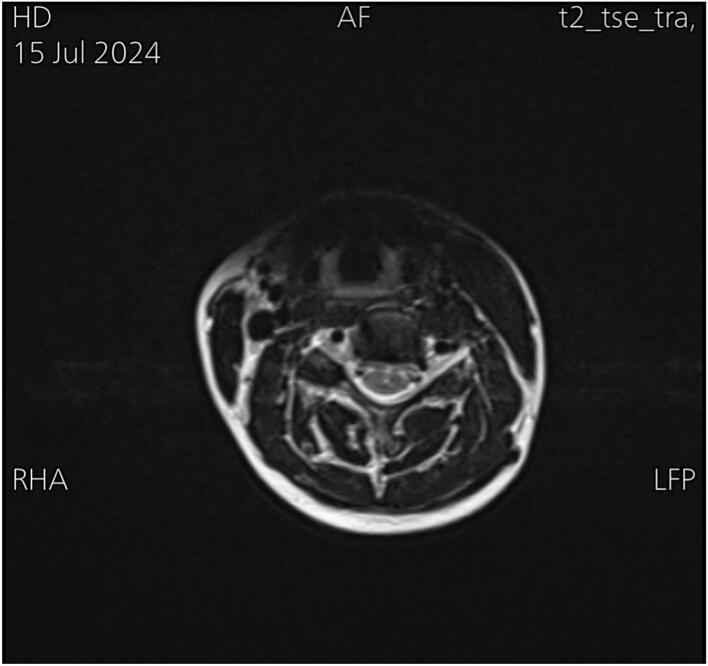
Axial T2-weighted MRI scan of the neck showing relative atrophy of the right sided sternocleidomastoid muscle with comparison to the left.

**Fig. 5:**
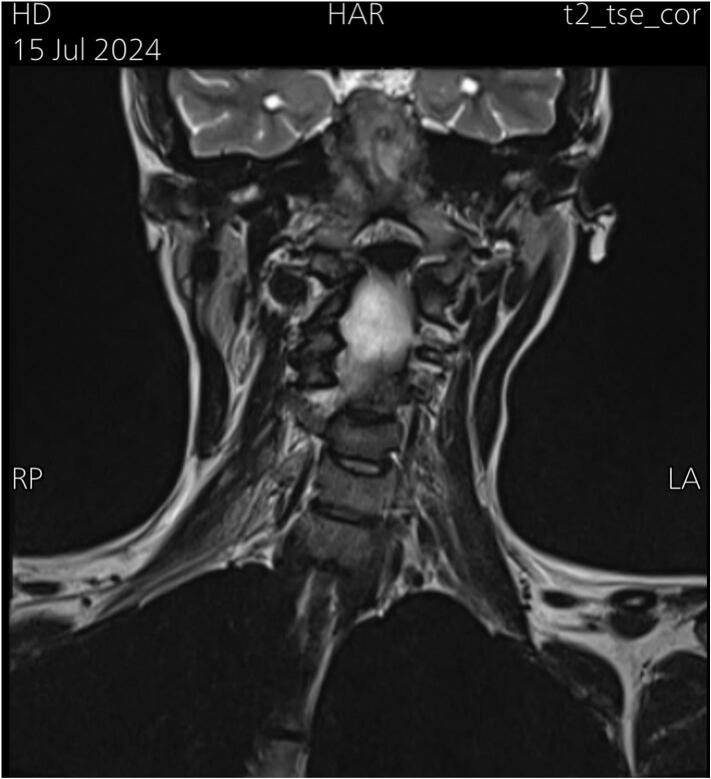
Coronal T2-weighted MRI scan of the neck showing moderate to severe rightward deviation of the lower cervical and visualized upper thoracic spine.

On physical examination, she had muscle tightness on the right side, with a left-sided rotational deficit of 12 degrees and lateral flexion deficit exceeding 15 degrees. A firm, cord-like structure was palpated in the lower right sternocleidomastoid muscle (SCM). (Fig. 1) illustrates the preoperative facial asymmetry and the cord-like structure observed on the right side. MRI showed a rightward deviation of the lower cervical and upper thoracic spine (Fig. 2), as well as atrophy of the right sternocleidomastoid muscle (SCM) (Fig. 3). Axial T2-weighted MRI scan of the neck showed relative atrophy of the right-sided sternocleidomastoid muscle compared to the left. (Fig. 4) Coronal T2-weighted MRI scan of the neck showed moderate to severe rightward deviation of the lower cervical and visualized upper thoracic spine (Fig. 5).

The patient underwent surgery by an orthopedic and peripheral hand surgeon. The sternal head of the right-sided sternocleidomastoid muscle (SCM) was resected under general anesthesia, with local anesthesia administered postoperatively. The surgical approach was right-sided supraclavicular, utilizing upper limb surgical instruments. Bipolar hemostasis was employed to prevent brachial plexus irritation. Initially, the plan was to prepare for bipolar resection; however, a decision was made intraoperatively to perform unipolar resection, as this approach was sufficient to achieve the necessary muscle correction after gentle manipulation. Resection of the right sternocleidomastoid (SCM) sternal head was performed up to 2 cm from the origin, and the clavicular head was found to be absent intraoperatively. The wound was closed with subcutaneous and subcuticular sutures for cosmetic purposes, and no drain was used. Postoperatively, there were no complications, except for mild erythema at the surgical site. Wound care included Non-steroidal anti-inflammatory drugs (NSAIDs), antibiotics (amoxicillin-clavulanic acid) for three days, and heparin sodium cream for scar resolution.

A cervical brace was worn for 20 days. Followed by a rehabilitation program commenced one month after surgery, with adherence to physiotherapy closely monitored by a doctor from the Hebron Rehabilitation Society. The exercises focused on improving range of motion and flexibility, including head movements in all directions (up and down, left and right) to the maximum extent. Physiotherapy sessions were held twice a week for a total of six sessions, with additional home exercises performed five times a day, each exercise repeated ten times. Initially, the exercises caused pain, discomfort, and mental exhaustion. However, over time, with continued practice, the pain significantly decreased and was rarely experienced during exercises. Notable improvements in muscle movement were observed. The patient was advised to continue the exercises for an additional six months. At this point, no further improvements were noted, suggesting that the final outcome had been achieved. Improvements in neck rotation and lateral flexion, and facial asymmetry resolved.

By three months post-surgery, neck motion deficits were reduced to less than 5 degrees. Functional and cosmetic outcomes were assessed using the Cheng and Tang scoring system, with the patient's preoperative score of 10 improving to 19 postoperatively as in Table (1). The patient expressed excellent satisfaction with the results. Fig. 4 provides a comparison of the sternocleidomastoid muscle (SCM) muscle preoperatively and 3 months postoperatively.

**Table 1:** 

Assessment	Preoperative	Postoperative (3 months)
Rotational deficit	1	3
Lateral flexion deficit	0	3
Craniofacial asymmetry	2	3
Scar	3	2
Residual contracture	1	2
Subjective assessment (cosmetic and functional)	2	3
Head tilt	1	3
Overall score	10	19

As a summary, the patient was diagnosed with congenital torticollis three years ago and underwent physiotherapy and dry needling without significant improvement. After deciding to undergo surgery, she had a successful procedure followed by six months of rehabilitation. The final outcome was achieved, with improvements in neck motion and facial asymmetry, and the patient reported high satisfaction with the results.

## 3 Discussion

Congenital Muscular Torticollis is a common pediatric musculoskeletal condition, characterized by a postural neck deformity, often evident at birth or shortly thereafter. It is defined by a head tilt or lateral neck flexion, with neck rotation to the opposite side, due to unilateral shortening or fibrosis of the SCM muscle [8]. Most infants with CMT are effectively managed with physical therapy [4]. If untreated, CMT can lead to complications such as pain, spinal deformities, and craniofacial irregularities [5,6]. Early intervention, including stretching exercises, physiotherapy, and botulinum toxin injections, is typically effective [9,10], while surgical options are reserved for severe or persistent cases [9].

This case highlights the complexities of managing CMT in adolescents and adults, where non-surgical treatments, including rehabilitation and orthosis, are generally ineffective [11]. In this case, surgical resection of the sternal head of the SCM muscle was performed, targeting areas with significant tension and atrophy. This approach aligns with established strategies for managing late-presenting CMT, such as unipolar or bipolar release, Z-plasty, or partial muscle resection [12,4]. Bipolar release is often the treatment of choice for neglected or recurrent cases in patients over six years old [9,13].

Unipolar resection is less commonly studied than release techniques, and there appears to be a lack of research comparing unilateral versus bilateral resection procedures directly. The unipolar resection performed in this patient resulted in adequate cervical spine mobilization, consistent with findings from a study that demonstrated unipolar release can yield outcomes comparable to bipolar release in adolescent or adult patients with neglected CMT. The study emphasized the importance of dissecting the lower portion of the SCM muscle until sufficient mobility is achieved. Additionally, unipolar release is less invasive, carries a lower risk of injury to the great auricular nerve, and may reduce wound size, operative time, and blood loss [5].

Although a cord-like structure was palpated in the right SCM, MRI findings showed no evidence of fibrosis or mass lesions, suggesting that the condition was likely due to muscle contracture or thickening instead. Moreover, imaging revealed moderate to severe rightward deviation of the lower cervical and upper thoracic spine, reflecting compensatory postural changes due to muscle imbalance, which is typical in CMT. This deviation did not require surgical correction. Furthermore, the atrophy of the right SCM muscle indicated long-standing dysfunction, contributing to the patient's abnormal head posture and restricted neck mobility. Atrophy could also be a sign of an absent clavicular head, which was observed intraoperatively; however, this absence did not affect the surgical approach or outcome.

Rehabilitation played a crucial role in the patient's recovery, with substantial mobility gains within three months. The patient's deficits in rotation and lateral flexion were reduced to less than 5 degrees, reflecting the success of the surgical and rehabilitation plan. Literature supports the importance of early physiotherapy and regular follow-up for optimizing outcomes in CMT [14].

While data on outcomes in adults with neglected CMT are limited, available reports suggest that, although challenging, intervention can still lead to positive results [15]. This underscores the importance of clinical awareness and early intervention whenever possible, as late treatment requires careful consideration of the associated complexities.

## 4 Conclusion

This case demonstrates that late-presenting CMT can still be effectively managed with surgical intervention and rehabilitation, even in adults. The use of unilateral SCM resection proved successful in addressing both functional and cosmetic concerns. Anatomical variations, such as the absence of the clavicular head of the SCM, must be considered when planning surgery. Postoperative rehabilitation played a crucial role in achieving significant improvements in neck mobility and facial symmetry. While challenging, timely intervention in neglected CMT cases can lead to favorable outcomes, highlighting the importance of early diagnosis and individualized treatment approaches.

## Informed consent

Written informed consent was obtained from the patient for publication of this case report and accompanying images. A copy of the written consent is available for review by the Editor-in-Chief of this journal on request.

## Ethical approval

Ethical approval was not applicable for this study, as our institution's IRB committee at Hebron University does not mandate approval for reporting individual cases or case series.

## Funding

This research did not receive any specific grants from funding agencies in the public, commercial, or not-for-profit sectors.

## Author contribution

S.A. handled conceptualization, data curation, and the writing of the original draft. R.A. contributed to writing, review, and editing. H.A. was responsible for investigation and visualization. Y.B. managed resources and validation. M.S. provided supervision and overall guidance.

## Guarantor

Roba Alzuhoor is the guarantor for this study, taking full responsibility for the research and its outcomes. Roba Alzuhoor had access to all the data and made the final decision to publish the study.

## Research registration number

N/A.

## Conflict of interest statement

The authors assert that no conflicts of interest exist in relation to this work.
